# Integrating artificial intelligence with human reasoning in oncology: questions on real-world implementation and patient-centric evidence

**DOI:** 10.1186/s40779-025-00663-7

**Published:** 2025-11-04

**Authors:** Carlos M. Ardila, Anny Marcela Vivares-Builes, Eliana Pineda-Vélez

**Affiliations:** 1 Department of Periodontics, Saveetha Dental College, and Hospitals, Saveetha Institute of Medical and Technical Sciences, Saveetha University, Saveetha, 600077 India; 2 Basic Sciences Department, Biomedical Stomatology Research Group, Faculty of Dentistry, Universidad de Antioquia U de A, 050010 Medellín, Colombia; 3 Faculty of Dentistry, Institución Universitaria Visión de las Américas, 050040 Medellín, Colombia

**Keywords:** Artificial intelligence (AI), Precision medicine, Standard of care

The article by Jiang et al. [1], “Leveraging artificial intelligence for clinical decision support in personalized standard regimen recommendation for cancer” published in *Military Medical Research*, addresses a pivotal issue in contemporary oncology: how artificial intelligence (AI) can augment clinical reasoning to refine regimen selection. Their discussion of data learnability, model usability, and the envisioned SINGULARITY framework reflects a forward-looking approach to precision medicine. The integration of real-world evidence into multimodal AI is indeed a necessary evolution toward context-aware decision support systems. Nevertheless, several important questions emerge from their proposal that may further enrich the dialogue on AI-guided oncology.

While the authors underscore the limitations of static and cross-sectional data in existing AI models, the question remains how temporal dynamics, such as treatment response trajectories, clonal evolution, or changing comorbidities, will be represented. Longitudinal modeling requires harmonized, repeated measures across diverse modalities, yet electronic health records and omic repositories are often incomplete or asynchronous [2]. How might multimodal systems reconcile these temporal mismatches without introducing bias or losing clinical interpretability? Moreover, in adaptive oncology, where therapy sequences are continually adjusted, can AI truly mirror the nuanced reasoning by which clinicians weigh prior outcomes, toxicity, and patient tolerance?

The SINGULARITY study proposes the use of “real-world data adhering to rigorous standards of clinical trials”. This hybrid approach raises methodological questions. Real-world data, by definition, lack the controlled assignment and homogeneity of trial settings [3]. How will the study account for confounders, missing data, and variations in data provenance when constructing the AI model? Will causal inference frameworks, such as target trial emulation or inverse probability weighting, be integrated to preserve validity while leveraging observational inputs? Without such safeguards, there is a risk that the abundant real-world heterogeneity could compromise causal clarity and reproducibility.

Equally compelling is the issue of explainability. The authors note that AI should act as an adjunct to clinicians, offering reasoning beyond human perception. Yet as models become more complex, interpretability often declines. How will the SINGULARITY system ensure that its recommendations are transparent enough to be trusted, contested, or refined by oncologists? Explainable AI methods, such as attention maps, feature attribution, or counterfactual reasoning, might be indispensable, but their alignment with regulatory frameworks for clinical decision support remains uncertain [4]. Can the model’s reasoning be made sufficiently intelligible for audit, accountability, and patient consent?

Another area inviting exploration concerns equity and generalizability. The authors rightly acknowledge the challenge of data accessibility and the need for equitable AI. However, large national datasets may inadvertently reflect the sociodemographic and genomic landscape of a single population. How will the system adapt to other healthcare contexts, ethnic backgrounds, and economic settings where disease profiles, treatment access, and patient preferences diverge? The promise of “personalized standard therapy” might falter if the underlying data fail to capture population diversity. Are federated learning or transfer learning strategies being considered to ensure inclusiveness without breaching data privacy?

The integration of patient values and priorities is one of the most forward-looking aspects of the proposed model. Yet it invites questions on operationalization. How are subjective factors such as quality-of-life goals, risk tolerance, and cultural attitudes toward aggressive care quantified and encoded into an AI framework? If patients’ stated preferences evolve during treatment, can the system dynamically update recommendations, or will it depend on static inputs? These issues underscore the broader challenge of embedding empathy, autonomy, and shared decision-making within algorithmic reasoning.

Furthermore, as generative AI and large language models become part of the clinical ecosystem, governance and validation frameworks remain in flux. Jiang et al. [1] highlighted that even when large language models displayed superior reasoning in controlled experiments, their clinical efficacy was uncertain. This leads to a practical question: what constitutes adequate evidence for integrating such systems into treatment recommendations? Should we rely on model-level validation (e.g., cross-validation, external test sets) or prospective human-AI co-performance trials demonstrating real-world benefit? The distinction between statistical accuracy and clinical utility is subtle but consequential.

Finally, the SINGULARITY design aims to bridge guidelines and personalization by generating optimized options “based on current guidelines”. However, as guidelines themselves evolve, how will the AI platform maintain synchronicity with rapidly updating evidence? Continuous learning systems risk propagating outdated rules or misinterpreting conflicting recommendations from different societies [5]. What safeguards will prevent model drift and ensure alignment with the most recent, peer-reviewed consensus? Transparency in update cycles and version control may prove as critical as algorithmic performance.

In sum, the contribution of Jiang et al. [1] represents an important step toward redefining how oncologists and intelligent systems collaborate in clinical decision-making. The questions above are offered not as critiques but as invitations to further clarify how data integrity, temporal modeling, interpretability, and patient-centric ethics will shape the next generation of AI-guided oncology frameworks. As technology moves from concept to clinical interface, these dimensions will determine whether AI fulfills its potential not merely to recommend, but to reason responsibly alongside human judgment.

## Data Availability

Not applicable.

## Abbreviation

AI: Artificial intelligence
